# Quantification of Nineteen Bioactive Components in the Ancient Classical Chinese Medicine Formula of Wen-Dan Decoction and Its Commercial Preparations by UHPLC-QQQ-MS/MS

**DOI:** 10.3390/molecules24112031

**Published:** 2019-05-28

**Authors:** Bing Zhang, Dongli Qi, Xiuping Deng, Zhe Ma, Yumei Wu, Zhifeng Xue, Dereje Kebebe, Peng Lu, Jiaxin Pi, Pan Guo, Nan Li, Zhidong Liu

**Affiliations:** 1 Tianjin State Key Laboratory of Modern Chinese Medicine, Tianjin University of Traditional Chinese Medicine, Tianjin 301600, China; zhangbing2018@gmail.com (B.Z.); dengxiuping163@163.com (X.D.); 18233181543@163.com (Z.M.); wym91116@163.com (Y.W.); xzhifeng2012@163.com (Z.X.); dereje.keborg@gmail.com (D.K.); lylupeng@163.com (P.L.); ppstar9999@sina.com (J.P.); gpandap@163.com (P.G.); linan20080402@163.com (N.L.); 2 The Institute of Traditional Chinese Medicine, Tianjin University of Traditional Chinese Medicine, Tianjin 301600, China; 3 Engineering Research Center of Modern Chinese Medicine Discovery and Preparation Technique, Ministry of Education, Tianjin University of Traditional Chinese Medicine, Tianjin 301600, China; 4 School of Pharmacy, Institute of Health Sciences, Jimma University, P.O. Box 378, Jimma, Ethiopia

**Keywords:** quantitative analysis, UHPLC-QQQ-MS/MS, Wen-Dan Decoction, commercial preparations

## Abstract

A UHPLC-QQQ-MS/MS method was developed to quantify the significant constituents in Wen-Dan Decoction (WDD), a traditional Chinese medicine. Analysis of 19 compounds was conducted on an ACQUITY UPLC^®^ BEH C18 Column (2.1 × 50 mm, 1.7 μm) using elution with a gradient elution of acetonitrile and 0.05% (*v*/*v*) formic acid in water. A triple quadrupole mass spectrometer was operated in negative ionization mode and positive ionization mode by multiple reaction monitoring (MRM), respectively. All calibration curves showed acceptable linearity (r ≥ 0.9950). The RSDs of intra- and inter-day precisions of low, mid and high concentrations were ≤ 8.88%. The repeatabilities (RSDs ≤ 7.17%) and stabilities (RSD ≤ 4.79%) of the samples were qualified. The recoveries were found in the range of 93.07 ± 3.86 to 103.98 ± 2.98% with the RSD varying between 1.30 and 7.86%. The final rapid, sensitive, precise, accurate and reliable UHPLC-QQQ-MS/MS method was used for the simultaneous quantification of 19 constituents in WDD and its commercial preparations. The strategy of combining the contents of the 19 chemicals in a daily dose of the WDD preparations with the hierarchical cluster analysis and the 3D principal component analysis was employed to effectively distinguish the WDD preparations provided by the different suppliers, which represents a contribution to the evaluation and control of the quality of WDD (or other decoctions consisting of the same herbs) and the preparations of WDD in other dosage forms such as tablets and granules.

## 1. Introduction

Wen-Dan Decoction (WDD), a Chinese medicine prescription, is formulated from Pinelliae Rhizoma, Bambusae Caulis in Taenias, Aurantii Fructus Immaturus, Citri Reticulatae Pericarpium, Glycyrrhizae Radix Et Rhizoma, Poria, Zingiberis Rhizoma and Jujubae Fructus, which are all included in the Chinese Pharmacopoeia (Version 2015) [1]. In April 2018, National Administration of Traditional Chinese Medicine of People’s Republic of China launched the “Ancient Classical Chinese Medicine Formula Catalogue (First edition)” notice containing WDD providing the basis for the second-development of WDD (http://kjs.satcm.gov.cn/zhengcewenjian/2018-04-16/7107.html). In China, WDD is clinically used for the treatment of insomnia, asthenia, dizziness, gastritis, ischemic stroke, Meniere’s disease, metabolic syndrome, depression and schizophrenia [2]. Moreover, in the past decades, it had been reported that WDD had pharmacological effects in the treatment of Alzheimer’s disease, negative emotions of insomnia, cardiac fibrosis, disorders of lipid metabolism, etc [3,4]. Although the clinical applications and pharmacological effects of WDD have been well explored, chemical composition analysis of WDD is still scant. For example, only a small number of the chemical constituents in WDD such as liquiritin, naringin, hesperidin and glycyrrhizic acid were quantified by Xu et al., 6-gingerol in WDD was determined by Li et al. and the fingerprint of WDD was reported by Wang et al. [5,6,7]. The development and application of mass spectrometry provides a quick and convenient method for the identification and quantification of contents in complex natural medicine extracts. For instance, Li et al. utilized UHPLC-MS for the identification and quantification of chemical constituents in the traditional Chinese medicinal formula Qi-Fu-Yin [8].

**Figure 1:**
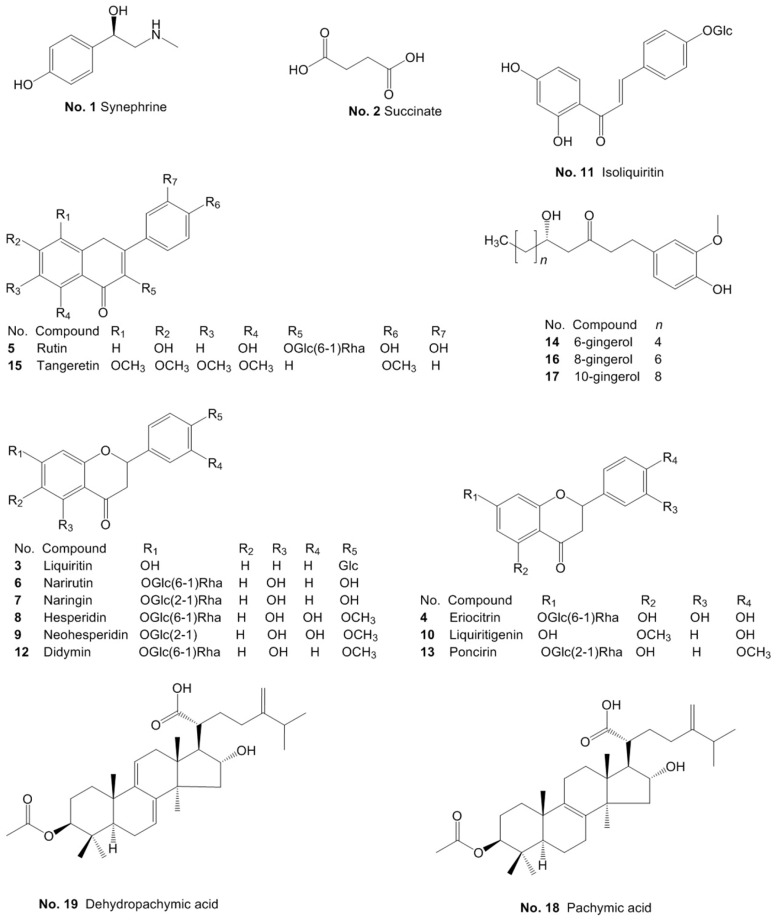
Structures of 19 chemicals analyzed in WDD.

In this study, an ultra-performance liquid chromatography method combined with triple quadrupole mass spectrometry (UHPLC-QQQ-MS/MS) was employed to identify and quantify some significant constituents in WDD. A total of 19 compounds, namely one organic acid (succinate), one alkaloid (synephrine), two triterpenoids (pachymic acid and dehydropachymic acid), three phenols (6-gingerol, 8-gingerol and 10-gingerol) and 12 flavonoids (rutin, eriocitrin, liquiritin, isoliquiritin, iosnaringin, naringin, hesperidin, neohesperidin, liquiritigenin, didymin, poncirin and tangeretin) were rapidly identified by the retention time and the MS/MS spectra data in both negative and positive ion modes. The structures of the 19 chemicals are shown in Figure 1. The contents of the ingredients were determined by comparison with reference substances of known purities. Finally, a reliable and validated UHPLC-QQQ-MS/MS method was developed for the content determination of 19 compounds in WDD and its marketed preparations. Fick’s first law of diffusion, differences between the extraction equipment and the dosage forms were utilized to account for the content variation among batches of traditional Chinese herb preparations and its marketed preparations. Furthermore, hierarchical cluster analysis and 3D principal component analysis were used to effectively distinguish the source of the preparations with combining the contents of the 19 components in WDD in a daily dose. The radar charts of the “total daily dosage of measured ingredients” of the seven pieces of traditional Chinese medicine and the radar charts of the 19 chemicals contents in a daily dose could help the researchers and doctors to make a better choice of the source of the WDD when they were going to use WDD. This study will contribute to the quality control, pharmacological research and clinical application of WDD and its commercial preparations.

## 2. Results

### 2.1. Optimization of LC Conditions

A gradient elution chromatographic procedure was used to achieve chromatographic separation of the 19 components in a short period of time. An ACQUITY UPLC^®^ BEH C18 column (1.7 μm, 2.1 × 50 mm) was employed for better peak symmetries than others, and acetonitrile was applied for its better chromatographic separation than that of methanol. The optimized column temperature of 35 °C and flow rate of 0.3 mL·min^−1^ were also contributed to the efficient separation. The 0.05% formic acid-water solution provided better peak shapes. After these optimization procedures, all reference compounds could be generally and chromatographically separated within 30 min.

### 2.2. Optimization of MS Conditions

**Figure 2:**
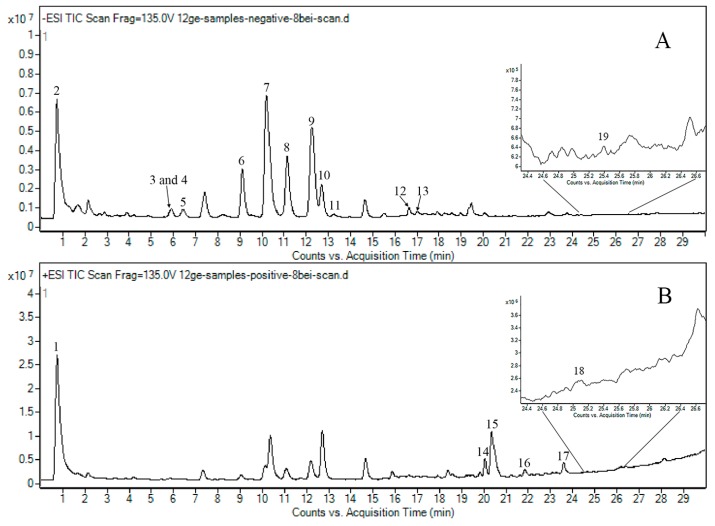
Total ion current scan chromatogram of negative ion (**A**) and total ion current scan chromatogram of positive ion (**B**) shows the main ingredients in the WDD sample, which are generally covered by the selected 19 chemicals. The substances corresponding to the numbers are the same as Figure 1, as follows: synephrine (**1**), succinate (**2**), liquiritin (**3**), eriocitrin (**4**), rutin (**5**), narirutin (**6**), naringin (**7**), hesperidin (**8**), neohesperidin (**9**), liquiritigenin (**10**), isoliquiritin (**11**), didymin (**12**), poncirin (**13**), 6-gingerol (**14**), tangeretin (**15**), 8-gingerol (**16**), 10-gingerol (**17**), pachymic acid (**18**) and dehydropachymic acid (**19**).

**Figure 3:**
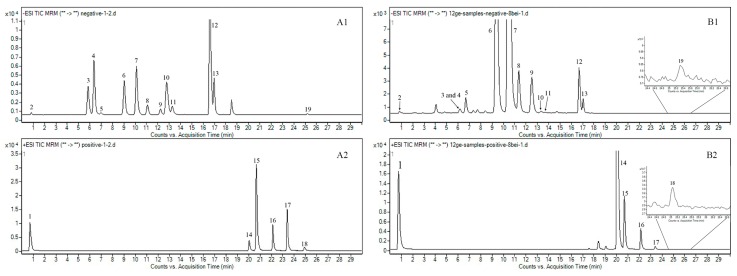
Total ion chromatograms in negative mode of the standard solutions (**A1**) and WDD sample (**B1**); total ion chromatograms in positive mode of the standard solutions (**A2**) and WDD sample (**B2**). The substances corresponding to the numbers are as follows: synephrine (**1**), succinate (**2**), liquiritin (**3**), eriocitrin (**4**), rutin (**5**), narirutin (**6**), naringin (**7**), hesperidin (**8**), neohesperidin (**9**), liquiritigenin (**10**), isoliquiritin (**11**), didymin (**12**), poncirin (**13**), 6-gingerol (**14**), tangeretin (**15**), 8-gingerol (**16**), 10-gingerol (**17**), pachymic acid (**18**) and dehydropachymic acid (**19**).

Studies on the individual mass spectra of the 19 compounds were performed by injecting the corresponding reference solutions into the mass spectrometer in both positive and negative ion modes. The results of the pilot study showed that the product ion peaks of succinate, liquiritin, eriocitrin, rutin, narirutin, naringin, hesperidin, neohesperidin, liquiritigenin, isoliquiritin, didymin, poncirin and dehydropachymic acid performed well in negative ion modes. Meanwhile, synephrine, 6-gingerol, tangeretin, 8-gingerol, 10-gingerol and pachymic acid gave better product ion results in positive ions mode. The fragmentor voltage (FV, 50–350V) and collision energy (CE, 0–50V) of all ingredients were optimized for greater abundances of precursor and product ion on the mass spectrometer, and the retention times were also determined by the reference solutions. The negative ion MS scan chromatogram (Figure 2A) and positive ion MS scan chromatogram (Figure 2B) show the main ingredients in the WDD sample, which are generally covered by the selected 19 chemicals. The total ion chromatograms in both negative and positive modes of the references (Figure 3A1,A2) and WDD sample solutions (Figure 3B1,B2) are shown in Figure 3. The optimized outcomes (formula, precursor ion, product ion, FV and CE) are listed in Table 1. The MRM chromatograms of 19 standard chemicals are shown in Supplementary Figure S1 and the characteristic product ion maps of the 19 chemicals are shown in Supplementary Figure S2.

### 2.3. Method Validation

The LODs and the LOQs of each compound showed acceptable sensitivities for the assays. The regression equations were constructed by comparing peak areas (Y) versus the concentrations (X) to present the linearity and the r ≥ 0.9950 demonstrated acceptable correlation coefficients for the calibration curves. The RSDs of intra-day precision (≤7.85%) and inter-day precision (≤8.88%) at three concentration levels (low, mid and high) indicated an acceptable precision of the method. The repeatabilities were found with RSDs of ≤7.17%. The components of the sample were stable (RSDs ≤ 4.79%) for 24 h at 4 °C in the autosampler after preparation, which is the acceptable stability during the testing process. The recoveries varied in the range from 93.07 ± 3.86 to 103.98 ± 2.98% with the RSDs ≤ 7.86%. All the results of the method validation mentioned above are summarized in Table 2 and Table 3. 

### 2.4. Quantification and Analysis of 19 Compounds in WDD and Its Commercial Preparations

Since the contents of the 19 components covered an extensive range, it was convenient for the establishment of the standard curves to dilute the WDD samples to different appropriate concentrations. Twelve replicates of WDD extracted in the lab and its five brands of commercial preparations were diluted according to the method mentioned in Section 4.4 and determined for the contents of 19 components by the validated UHPLC-QQQ-MS/MS method in 30 min. The assay efficiency had significant improvements over the HPLC-based investigation, ensuring that we could test more samples in a day.

**Figure 4:**
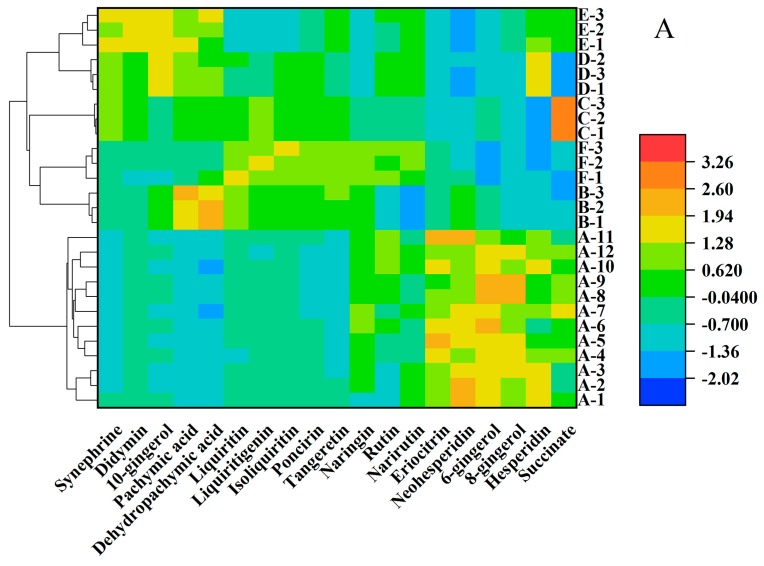

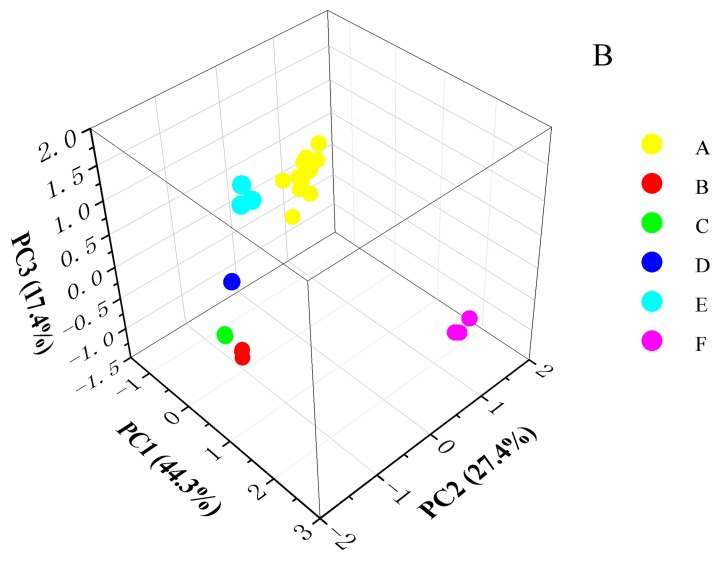
Heat map and hierarchical cluster analysis (**A**) and 3D principal component analysis (**B**) of the 19 chemicals contents in a daily dose of WDD prepared in the lab and its five brands commercial preparations. The contents results were normalized by the z-score method by OriginPro 2018 software and the raw data is provided by Table 4. A: WDD prepared in the lab; B: WDD from Kotaro Pharmaceutical Co., Ltd; C: WDD from Sheng Foong Co., Ltd; D: WDD (concentrated particles) from Kaiser Pharmaceutical Co., Ltd; E: WDD (concentrated ingots) from Kaiser Pharmaceutical Co., Ltd; F: WDD from Sun Ten Pharmaceutical Co., Ltd.

The range, the mean and standard deviation of the 19 chemical contents in a daily dose of WDD prepared in the lab and its five brands commercial preparations are shown in Table 4 and the raw data of the chemical contents in a daily dose of WDD prepared in the lab and its five brands commercial preparations are shown in Supplementary Table S1. Among the investigated substances in the 12 batches of WDD, 6-gingerol was the most abundant contents in a daily dose for the most proportion of Zingiberis Rhizoma (15.5 g). The two triterpenoids pachymic acid and dehydropachymic acid were not detected in the WDD samples prepared in the lab. Three flavonoids (liquiritin, isoliquiritin and liquiritigenin) in glycyrrhizae radix et rhizoma provided the larger content RSDs in the 12 batches WDD. The contents of 19 components in a daily dose of the 12 batches of WDD extracted in the lab and five brands WDD commercial preparations were normalized by z-score method. The normalized results were expressed in a heatmap and analyzed by hierarchical cluster analysis (Figure 4A) and 3D principal component analysis (Figure 4B). The corresponding results of the hierarchical cluster analysis and the 3D principal component analysis are about divided into five branches (A-1–A-3; A-4–A-6; A-7–A-9; A-10–A-12). The result almost matched the four decoction pots we used in the extraction process (Supplementary Figure S3). 

As shown in Figure 4A, the WDD prepared in the lab (A-1–A-12) and WDD commercial preparations (B-1–B-3; C-1–C-3; D-1–D-3; E-1–E-3; F-1–F-3) were classified into two clusters. The five commercial WDD formulations are then grouped into five different second-layer clusters, which matched their brands. Additionally, the two kinds of commercial WDD formulations from Kaiser Pharmaceutical Co., Ltd (concentrated particles of D-1–D-3 and concentrated ingots of E-1–E-3) were in one sort of cluster. As shown in Figure 4B, the contribution of PC1–PC3 was 89.1%. According to PCA analysis, the samples are classified into six groups according to their origins, which matched the sources of the WDD preparations.

## 3. Discussion

### 3.1. Selection of Indicators and Preparation of Solutions

Nineteen indicators, including one organic acid, one alkaloid, two triterpenoids, three phenolics and 12 flavonoids, were selected by reviewing previous reports and the Chinese Pharmacopeia. From the negative and positive scan chromatograms of the WDD sample (Figure 2A,B), it can be concluded that the selected 19 chemicals generally cover the main ingredients in WDD. 

Among the 19 chemicals, succinate, synephrine, naringin, didymin, 6-gingerol and pachymic acid feature a neuromodulation effect, and they are the major active constituents in WDD and responsible for the treatment of insomnia in clinical practice. Succinate was the active ingredient of Pinelliae Rhizoma [1]. Pachymic acid and dehydropachymic acid were obtained from Poria [9]. Rutin was extracted from Jujubae Fructus [10]. Liquiritin, Isoliquiritin and liquiritigenin were isolated from Glycyrrhizae Radix Et Rhizoma [11,12]. Synephrine, eriocitrin, narirutin, naringin, hesperidin, neohesperidin, didymin, poncirin and tangeretin were traced back to Aurantii Fructus Immaturus and Citri Reticulatae Pericarpium [1,13,14]. 6-Gingerol, 8-gingerol and 10-gingerol belong to Zingiberis Rhizoma [15,16]. These compounds covered an extensive range of polarities, so a 50% (*v*/*v*) methanol-water solution was used as the vehicle for good dissolutions of the different polar components. At the same time, the reference solutions were also prepared with the same vehicle to avoid the influence of different solvents.

### 3.2. Quantification and Analysis of 19 Compounds in WDD and Its Commercial Preparations

Among the investigated substances in the 12 batches of WDD, 6-gingerol was the most abundant component in a daily dose due to the larger proportion of Zingiberis Rhizoma (15.5 g) in the preparation. 6-Gingerol can reduce the level of dopamine in vivo to inhibit nerve activity [17], which can be employed to treat insomnia. With regard to 6-gingerol, although the daily dose is the largest among the 19 ingredients, we cannot ignore the potential loss of 6-gingerol during the process of extracting WDD in the lab. It has been reported that heating is adverse for the stability of 6-gingerol, which will rapidly be converted into the dehydration product shogaol at high temperature [18,19,20]. Moreover, organic solvents such as methanol, ethanol, isopropyl alcohol or *n*-hexane were generally used for better solubility of gingerol than water [16,21,22]. However, during the process of extracting WDD in the traditional way, the solvent (water) and boiling temperature (100 °C) are not conducive to the stability of gingerols. In addition, the extraction using a traditional decoction pot led to the loss of gingerols with the water vapor [23,24,25]. Additionally, compared with the GC-MS which requires heating samples, the UHPLC-MS is more beneficial for the stability of the gingerols in this study [16].

Two triterpenoids of pachymic acid and dehydropachymic acid were not detected in the WDD samples prepared in the lab. The low percentages of pachymic acid and dehydropachymic acid in Poria and their poor water-solubility partly accounted for their absence in the WDD samples prepared in the lab. Furthermore, their loss by the release of water vapor could reduce their contents in the WDD solution.

Three flavonoids (liquiritin, isoliquiritin and liquiritigenin) in glycyrrhizae radix et rhizoma provided the larger content RSDs in the 12 batches WDD. Fick’s first law of diffusion (1) was utilized to explore the sources of the large differences in the content RSDs of these three flavonoids in glycyrrhizae radix et rhizoma in the 12 batches WDD prepared in the lab [26,27]:

Ds = − DF dc/dx∙dt,(1)

where, dt is diffusion time (s). ds is the amount of diffused compound in dt time (mol). F is the diffusion area and represents the size and surface state of the herbs (m^2^). dc is the concentration difference of the compounds between the herbs and the solution (mol/m^3^). dx is the characteristic length scale of the diffusion system (m). D is the diffusion coefficient (m^2^/s). “-” indicates a decrease of concentration difference when the diffusion tends to equilibrium. According to Equation (1), the differences in the diffusion area and the concentration gradient are the main factors causing the variances in the content of ingredients in different batches of traditional Chinese medicine decoctions. In one dosage of the decoction, the surface area of the materials differs due to its size (shown in Supplementary Figure S4). Meanwhile, some reports have pointed out that growing region, harvest time, germplasm line, growing years and drying process gave rise to content variations of components between individual herbs [28,29,30,31,32]. Even in a single herb, significant differences in the amount of ingredients were discovered in different parts, such as the main root, branch root and fibrous root [33]. All of the above factors might give an explanation for the significant content difference between batches when the only 1.8 g of uneven glycyrrhizae radix et rhizoma was used in one dosage. The herb materials with different content of compounds also gave rise to the various content of ingredients among the batches of WDD.

The hierarchical cluster analysis results of the contents of 19 components in one daily dose in the 12 batches of WDD extracted in the lab almost matched the four decoction pots we used in the extraction process (Supplementary Figure S3), which suggest that even if we use the different types of equipment of the same type from the same manufacturer to extract the WDD, they would still lead to the variations in contents of the ingredients in the decoction procedures [34].

In the hierarchical cluster analysis and the 3D principal component analysis, it was found that the WDD granules (Figure 4D-1–D-3) and WDD ingots (Figure 4E-1–E-3) both acquired from Kaiser Pharmaceutical Co., Ltd. were divided into the one second-level cluster, which indicated their homology in regards to the materials and the extraction process. However, they were distinguished into two different first-level clusters instead of being mixed together in one, which implied that though the same WDD related preparations from one manufacturer, the daily dose of the ingredients might be distinct depending on the dosage form, which might lead to different therapeutic effects.

In general, the difference in a daily dose of the chemicals between the WDD prepared in the lab and WDD commercial preparations may be caused by the discrepancy of the ingredient contents in the herbs, the shape of the herbs, the size of the herbs, the divergence in the extraction method and equipment, the composition of the prescription (Supplementary Table S2) and the dosage form. Therefore, it is essential to assay the contents of the ingredients in herbal extraction related preparations before confirming the dosage quantity of herbal medicine for better treatment effects [16,35]. The uniform contents of compounds are apparently more conducive to the clinical application of traditional Chinese medicine preparations. In order to obtain batches of uniform contents decoctions or its related preparations, the proportions of various herbs, the contents of the ingredients, the sizes of the herbs and the extraction equipment should be controlled.

### 3.3. Recommendation on Preclinical Research and Clinical Application of WDD

**Figure 5:**
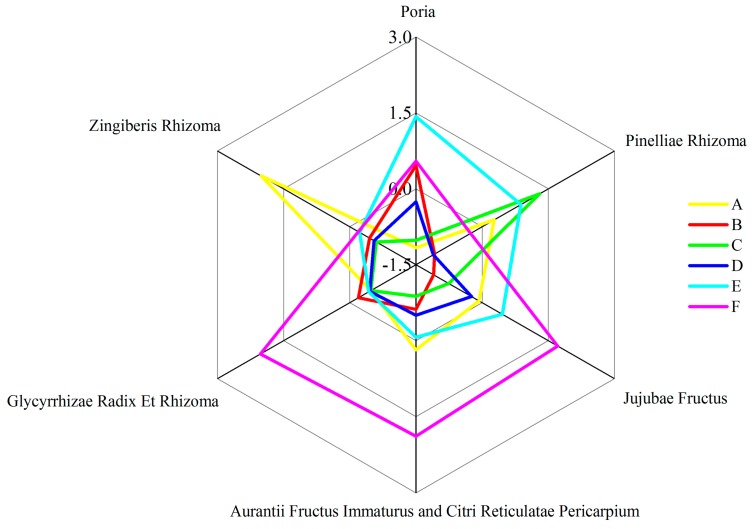
The radar chart of z-score normalized data of “total daily dosage of measured ingredients” of the seven pieces of traditional Chinese medicine in six WDD preparations. The daily dosage of Zingiberis Rhizoma is expressed as the sum of the mean daily dosages of 6-gingerol, 8-gingerol and 10-gingerol. The daily dosage of Pinelliae Rhizoma is revealed as the mean daily dosages of succinate. The daily dosage of Jujubae Fructus is illustrated as the mean daily dosages of rutin. The daily dosage of Glycyrrhizae Radix Et Rhizoma is demonstrated as the sum of the mean daily dosages of liquiritin, isoliquiritin and liquiritigenin. The daily dosage of Poria is displayed as the sum of the mean daily dosages of pachymic acid and dehydropachymic acid. The daily dosage of Aurantii Fructus Immaturus and Citri Reticulatae Pericarpium is shown as the sum of the mean daily dosages of synephrine, eriocitrin, narirutin, naringin, hesperidin, neohesperidin, didymin, poncirin and tangeretin.

In Section 3.1, it is pointed out that the 19 determined components have been originated from seven pieces of traditional Chinese medicine. Supplementary Table S3 displays the “total daily dosages of measured ingredient” for the seven pieces of traditional Chinese medicine, which is defined as the sum of the average daily dosages of all the measured ingredients tracking back to a certain piece of traditional Chinese medicine. For example, the daily dosage of Zingiberis Rhizoma is expressed as the sum of the mean daily dosages of 6-gingerol, 8-gingerol and 10-gingerol. The z-score normalized data of the “total daily dosage of measured ingredient” of the seven pieces of traditional Chinese medicine is represented by a radar chart showing seven pieces of traditional Chinese medicine from six different WDD preparations. These advantages and disadvantages can further provide a stroma for the selection of the source of WDD preparation, according to the patient condition in the clinical practice under the guidance of traditional Chinese medicine theory. For example, in traditional Chinese medicine theory, Zingiberis Rhizoma has a role in treating vomiting [1]. If the relevant conditions of insomnia and asthenia accompanied by vomiting are to be treated clinically, the WDD prepared in the lab (Figure 5A) can be given priority in the six WDD preparations involved in this study.

**Figure 6:**
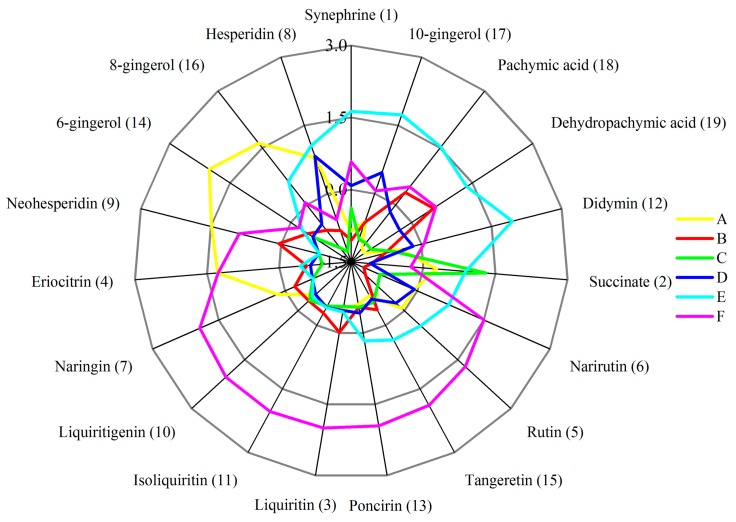
The radar chart of z-score normalized data of 19 chemicals contents in a daily dose of WDD prepared in the lab and its five brands commercial preparations. The average contents of the 19 chemicals in a daily dose of the six WDD preparations is provided in Table 4.

Furthermore, the average chemicals contents in a daily dose of WDD prepared in the lab and its five brands commercial preparations are shown in Table 4. The z-score normalized data of the average chemicals contents in a daily dose of the above WDD preparations is displayed in another radar chart (Figure 6), which can be used to preliminarily characterize the advantages and disadvantages of 19 chemicals in six different WDD preparations. These advantages and disadvantages provide a basis for the selection of the source of WDD preparation for preclinical research and clinical application based on the modern pharmacological research theory. Generally, before a single component of the 19 components of the measured WDD exerts its pharmacological activity, the concentration of component should reach an effective level, which is closely related to the daily dose of the component. It can contribute to the preclinical research and the clinical application by selecting the source of the WDD preparation based on selecting a certain chemical owning a required pharmacological activity and a superior average daily dosage in the six WDD preparations. For example, it is shown in Figure 6 that WDD prepared in the lab (Figure 6A) possesses the superiorities in the average daily dosage of 6-gingerol and 8-gingerol. As it is reported that the gingerols have the pharmacological activity in the treatment of vomiting [36], when it comes to the preclinical research of the antiemetic effect of WDD or the clinical application for the treatment of vomiting associated with insomnia, WDD prepared in the lab could be a better choice.

## 4. Materials and Methods 

### 4.1. Reagents and Materials

Reference substances of rutin (batch no.: W14-2-9), eriocitrin (batch no.: W18-2-3), liquiritin (batch no.: W10-4-8), isoliquiritin (batch no.: W14-8-5), narirutin (batch no.: W00-7-4), naringin (batch no.: W00-8-2), hesperidin (batch no.: W00-0-6), neohesperidin (batch no.: W00-7-6), liquiritigenin (batch no.: W13-7-2) and tangeretin (batch no.: W14-1-2) were supplied by Tianjin Zhongxin Pharmaceutical Group Co., Ltd. (Tianjin, China). Succinate (batch no.: H-059-170426), pachymic acid (batch no.: F-006-181210), dehydropachymic acid (batch no.: Q-073-181210), didymin (batch no.: X-034-171217), poncirin (batch no.: G-019-171216), 6-gingerol (batch no.: J-019-171128), 8-gingerol (batch no.: J-020-170517) and 10-gingerol (batch no.: J-037-170517) were purchased from Chengdu Ruifensi Biological Technology Co., Ltd. (Chengdu, China). Synephrine (batch no.: Y2607Y17088) was obtained from Shanghai Yuanye Bio-technology Co., Ltd. (Shanghai, China). The purities of all 19 abovementioned reference substances were no less than 98% and their chemical structures are shown in Figure 1. Acetonitrile and methanol (HPLC grade) were obtained from Thermo Fisher Scientific Inc. (Shanghai, China), and the formic acid was supplied by Tokyo Chemical Industry Co., Ltd. (LC-MS grade, Tokyo, Japan). The deionized water was produced by a Milli-Q water purification system (Millipore, MA, USA).

The herbal materials of Pinelliae Rhizoma (Si Chuan, China, batch no.: 160108), Bambusae Caulis in Taenias (Guang Xi, China, batch no.: 160108), Aurantii Fructus Immaturus (Zhe Jiang, China, batch no.: 160108), Citri Reticulatae Pericarpium (Si Chuan, China, batch no.: 160108), Glycyrrhizae Radix Et Rhizoma (Nei Meng, China, batch no.: 160108), Poria (Guang Xi, China, batch no.: 160108), Zingiberis Rhizoma (Si Chuan, China, batch no.: 160301) and Jujubae Fructus (Shan Dong, China, batch no.: 160108), which are all in compliance with the requirements described in the in Chinese Pharmacopoeia (Version 2015), were purchased from Anhui Yiyuantang Herbal Medicine Co., Ltd. (Anhui, China).

Essence fine particles of WDD (batch no.: YJ779) was bought from Kotaro Pharmaceutical Co., Ltd. (Osaka, Japan). Concentrated powder of WDD (batch no.: E085RQ1) was acquired from Sheng Foong Co., Ltd. (Taiwan, China). Concentrated particles of WDD (batch no.: K30222) was purchased from Kaiser Pharmaceutical Co., Ltd. (Taiwan, China). Concentrated ingots of WDD (batch no.: H05125) was obtained from Kaiser Pharmaceutical Co., Ltd. (Taiwan, China). Concentrated particles of WDD (batch no.: 18092733) was gained from Sun Ten Pharmaceutical Co., Ltd. (Taiwan, China). 

### 4.2. UHPLC-QQQ-MS/MS Conditions

The quantitative analysis was carried out on an Agilent 1290 series UHPLC-triple quadrupole mass spectrometry (UHPLC-QQQ-MS/MS) system (Agilent Technologies, Palo Alto, CA, USA). The chromatographic separation was performed on an ACQUITY UPLC^®^ BEH C18 column (1.7 μm, 2.1 × 50 mm, Waters, Milford, MA, USA) and the column temperature was maintained at 35 °C by a column thermostat. The autosampler temperature was controlled at 4 °C and the sample injection volume was 2 μL. A mixture of 0.05% (*v*/*v*) formic acid water (A) and acetonitrile (B) was used as the mobile phase, which was driven by a binary pump at a flow rate of 0.3 mL·min^−1^. The gradient elution program was as follows: 0–5 min, 10–13%B; 5–11 min, 13–17%B; 11–14 min, 17–19% B; 14–20 min, 19–47% B; 20–22 min, 47–67% B; 22–26 min, 67–80% B; 26–30 min, 80–90% B and the post time was set as 3 min. The UHPLC system was coupled to an Agilent 6460 triple quadrupole mass spectrometer (Agilent Technologies) equipped with an ESI source. Both negative ionization mode and positive ionization mode were used for the analysis and determination. The MS conditions were set as follows: capillary voltage at 3500 V; gas (N_2_) temperature at 350 °C; drying gas flow rate at 10 L·min^−1^; nebulizer gas (N_2_) pressure at 35psi; high purity nitrogen as the collision gas. The multiple reaction monitoring (MRM) was used for quantification. The ionization mode, fragmentor voltage, collision energy and the pair of precursor-product ions for each compound were optimized in the following study. A MassHunter Workstation software (Version B.07.00, Agilent Technologies) was employed for the LC-MS data acquisition and analysis. The acceptable accuracy variation between the measured concentrations and the actual value was set within 20%.

### 4.3. Preparation of Standard Solution

Synephrine, succinate, liquiritin, eriocitrin, rutin, narirutin, naringin, hesperidin, neohesperidin, liquiritigenin, isoliquiritin, didymin, poncirin, 6-gingerol, tangeretin, 8-gingerol, 10-gingerol, pachymic acid and dehydropachymic acid were accurately weighted on a XP205 micro-balance (Mettler Toledo, Zurich, Switzerland) and dissolved in 50% (*v*/*v*) methanol-water at suitable concentrations, respectively. All of the stock solutions were stored at 4 °C.

### 4.4. Preparation of Samples Solutions

Pinelliae Rhizoma (3.7 g), Bambusae Caulis in Taenias (3.7 g), Aurantii Fructus Immaturus (3.7 g), Citri Reticulatae Pericarpium (5.4 g), Glycyrrhizae Radix Et Rhizoma (1.8 g), Poria (2.7 g), Zingiberis Rhizoma (15.5 g) and Jujubae Fructus (2.5 g) were put in a decoction pot (2L, Kangyashun Electric Co., Ltd., Guangdong, China) and immersed in 300 mL deionized water for 30 min. The formulation was boiled at 220 V and then evaporated to 150 mL at 175 V. The remaining decoction was filtered with two layers of gauze to obtain one dose of WDD. Appropriate volume of WDD was mixed with the same volume of methanol by sonication (320 W, 40 kHz) with a KQ-400KDE ultrasonic cleaner (Kunshan Ultrasonic Instruments Co., Ltd., Jiangsu, China) for 10 min. Since it was assayed that 25 mL WDD we extracted in the lab included about 0.6685 g of dry-powder, the five brands of WDD commercial preparations were ground into uniform fine powders (composed of some excipients and the extracted dry-powder) and then different weights of the uniform fine powders (containing about 0.5 g extracted dry-powder) were dissolved in 25 mL deionized water, respectively, and then they were mixed with the same volume of methanol by sonication (320 W, 40 kHz) with the KQ-400KDE ultrasonic cleaner for 10 min.

The above six mixtures were centrifuged (10,000 rpm, 4 °C) with a Sorvall ST16R centrifuge (Thermo Fisher Scientific Inc., Shanghai, China) for 10 min to obtain the supernatants. The supernatants were diluted four times by 50% (*v*/*v*) methanol-water for the determination of synephrine, succinate, liquiritin, eriocitrin, rutin, liquiritigenin, isoliquiritin, didymin, poncirin, tangeretin, 8-gingerol, 10-gingerol, pachymic acid and dehydropachymic acid. Similarly, the supernatants were diluted 40 times by 50% (*v*/*v*) methanol-water for the determination of narirutin, naringin, hesperidin, neohesperidin, and 6-gingerol. All the samples were filtered through a 0.22 μm microporous membrane before the determination.

### 4.5. Method Validation

The 19 stock solutions were precisely mixed and further diluted with 50% (*v*/*v*) methanol-water to prepare the calibration curves owing different concentration ranges. All calibration curves were established by the ratio of standard reference peak areas (Y) versus their concentrations (X) with the weight of 1/X.

Limit of detection (LOD, signal-to-noise values (S/N) ≥3) and limit of quantification (LOQ, signal-to-noise values (S/N) ≥10) of the 19 chemicals were obtained by determining continuously diluted standard mixture to assess the sensitivity. The stabilities of the compositions were detected by assaying the prepared samples stored at 4 °C for 0, 2, 4, 6, 8, 10, 12 and 24 h. Intra-day (1 day) and inter-day (3 consecutive days) precisions at low, mid and high concentration levels within the calibration curves were examined. Replicates (n = 6 for diluted 4 times and 40 times, respectively) of WDD samples were prepared as the method in Section 2.4 and determined to demonstrate the repeatability. Relative standard deviation (RSD) was employed to evaluate the stabilities, precisions, and repeatabilities.

The recovery was surveyed to evaluate the method accuracy. The spiked samples were prepared by mixing known amounts of the 19 standard references with the known amounts of quantitatively analyzed WDD samples in sextuplicate, and then diluted and analyzed with the same procedures.

### 4.6. Statistics Analysis

The data were presented as “mean ± S.D.”. The heatmap-dendrogram application from OriginPro 2018C (v9.5.1) software was employed for the hierarchical cluster analysis and the demonstration of the daily dose of 19 compounds in WDD samples and its commercial preparations. The 3D principal component analysis application from OriginPro 2018C software was used for the 3D principal component analysis of 19 compounds in WDD samples and its commercial preparations. The radar chart application from OriginPro 2018C software was utilized for the comparisons of the total daily dosage of the determined ingredients in the respective traditional Chinese medicines and the daily dosage of each the 19 ingredients in the six WDD preparations.

## 5. Conclusions

In this study, a validated and reliable UHPLC-QQQ-MS/MS method was developed for the simultaneous quantification of 19 compounds in WDD and its commercial preparations, including one organic acid, one alkaloid, two triterpenoids, three phenols and 12 flavonoids. In addition, the proportions of various herbs, the contents of the ingredients in the herbs, the Fick’s first law of diffusion, the differences between the extraction equipment used and the dosage form were utilized explain for the content variations among batches of traditional Chinese herb preparations and its marketed preparations. Furthermore, it can be used to effectively distinguish the sources of the preparations with combining the contents of the 19 components in WDD in a daily dose with the hierarchical cluster analysis and 3D principal component analysis. Simultaneously, the developed method could contribute to the evaluation and control the quality of WDD (or other decoctions consisting of the same herbs) and the preparations of WDD in other dosage forms such as tablets and granules. The radar charts of the “total daily dosage of measured ingredients” of the seven pieces of traditional Chinese medicine and the 19 chemicals contents in a daily dosage would do some help to the development of the researches on preclinical research and clinical application of WDD.

## Figures and Tables

**Table 1:** Chemical formulas, masses, precursor ions, product ions, FVs and CEs of the 19 chemicals.

No.	Compound	Formula	Mass	Precursor Ion (*m*/*z*)	Product Ion (*m*/*z*)	FV (V)	CE (eV)
**1**	Synephrine	C_9_H_13_NO_2_	167.2	168.21 [M + H]^+^	149.9	46	8
**2**	Succinate	C_4_H_6_O_4_	118.1	117.1 [M − H]^−^	73.1	62	9
**3**	Liquiritin	C_21_H_22_O_9_	418.4	417.4 [M − H]^−^	254.9	184	14
**4**	Eriocitrin	C_27_H_32_O_15_	596	595 [M − H]^−^	287	290	21
**5**	Rutin	C_27_H_30_O_16_	610.4	609.4 [M − H]^−^	300.3	250	38
**6**	Narirutin	C_27_H_32_O_14_	580	579 [M − H]^−^	271	250	34
**7**	Naringin	C_27_H_32_O_14_	580	579 [M − H]^−^	271	250	34
**8**	Hesperidin	C_28_H_34_O_15_	610.5	609.5 [M − H]^−^	301	136	22
**9**	Neohesperidin	C_28_H_34_O_15_	610.5	609.5 [M − H]^−^	301	166	38
**10**	Liquiritigenin	C_15_H_12_O_4_	256.2	255.2 [M − H]^−^	119	130	21
**11**	Isoliquiritin	C_21_H_22_O_9_	418.4	417.4 [M − H]^−^	254.9	224	14
**12**	Didymin	C_28_H_34_O_14_	594	593 [M − H]^−^	285	290	29
**13**	Poncirin	C_28_H_34_O_14_	594	593 [M − H]^−^	285	270	38
**14**	6-Gingerol	C_17_H_26_O_4_	294	277 [M + H − H_2_O]^+^	177	80	5
**15**	Tangeretin	C_20_H_20_O_7_	372	373 [M + H]^+^	343	165	26
**16**	8-Gingerol	C_19_H_30_O_4_	322	305 [M + H − H_2_O)^+^	177	80	5
**17**	10-Gingerol	C_21_H_34_O_4_	350	333 [M + H − H_2_O]^+^	177	80	5
**18**	Pachymic acid	C_33_H_52_O_5_	528	528 [M − e]^+^	510.3	112	10
**19**	Dehydropachymic acid	C_33_H_50_O_5_	527	527 [M + e]^−^	465.3	224	45

**Table 2:** Regression data, LODs, LOQs, stabilities, repeatabilities and recoveries for the 19 components of WDD.

No.	Compound	Regression equation	r	LOD	LOQ	Linear Range	Stability	Repeatability	Recovery (%, n = 3)	
				(ng)	(ng)	(ng/mL)	(RSD, 24 h)	(RSD, n = 6)	mean ± S.D.	RSD
**1**	Synephrine	Y = 131.98 X	0.9957	0.3125	2.5000	2.5000–2560.0	3.17	3.02	98.41 ± 5.61	5.70
**2**	Succinate	Y = 1.9032 X	0.9972	12.500	100.00	100.00–1600.0	2.65	7.17	101.53 ± 6.61	6.51
**3**	Liquiritin	Y = 22.716 X + 38.196	0.9999	0.8292	6.6339	20.110–2574.5	2.51	2.08	98.47 ± 2.20	2.23
**4**	Eriocitrin	Y = 2.4774 X − 65.670	0.9995	1.7161	54.916	104.06–13320	4.15	4.43	101.98 ± 3.79	3.72
**5**	Rutin	Y = 2.2459 X − 46.415	0.9992	7.0153	56.122	101.62–14228	4.13	3.67	101.92 ± 4.78	4.69
**6**	Narirutin	Y = 6.9416 X + 129.04	1.0000	1.5076	12.061	96.460–24695	2.97	2.88	94.93 ± 7.47	7.86
**7**	Naringin	Y = 3.6762 X + 156.30	0.9995	6.7731	27.092	102.68–13141	4.01	1.87	93.98 ± 4.12	4.38
**8**	Hesperidin	Y = 8.3003 X + 141.43	0.9950	3.4741	27.793	105.33–13481	3.48	0.64	95.79 ± 2.14	2.24
**9**	Neohesperidin	Y = 4.3130 X + 51.717	0.9965	13.358	26.716	101.25–12960	1.73	0.95	98.17 ± 1.75	1.78
**10**	Liquiritigenin	Y = 7.1550 X + 4.0809	0.9996	0.4310	6.8955	10.450–1338.0	4.79	2.33	93.07 ± 3.86	4.15
**11**	Isoliquiritin	Y = 1.3129 X + 5.5881	0.9997	7.2305	57.844	109.61–14030	4.14	2.66	96.43 ± 1.73	1.80
**12**	Didymin	Y = 3.1519 X + 12.527	0.9998	3.4149	13.660	20.710–2650.5	3.38	4.05	97.97 ± 2.48	2.53
**13**	Poncirin	Y = 9.3281 X + 58.053	1.0000	0.7895	3.1580	12.630–3233.7	4.24	3.64	102.44 ± 4.75	4.63
**14**	6–Gingerol	Y = 24.238 X + 2249.5	0.9991	1.9739	3.9478	31.580–8085.0	2.65	1.86	100.30 ± 4.64	4.63
**15**	Tangeretin	Y = 251.42 X + 1050.1	0.9996	1.6582	6.6327	20.110–2574.0	4.31	2.55	103.98 ± 2.98	2.86
**16**	8–Gingerol	Y = 57.201 X + 121.47	1.0000	0.0270	0.1080	3.4600–884.50	2.73	3.16	100.22 ± 1.30	1.30
**17**	10–Gingerol	Y = 59.010 X + 38.030	0.9999	0.0315	0.1261	0.2522–64.560	3.80	3.55	95.76 ± 2.84	2.97
**18**	Pachymic acid	Y = 18.835 X	0.9997	1.7200	6.8600	6.8600–440.00	ND	ND	ND	ND
**19**	Dehydropachymic acid	Y = 2.3682 X	0.9997	1.5600	6.2500	6.2500–200.00	ND	ND	ND	ND

ND: not detected.

**Table 3:** Precisions of the 19 chemicals.

No.	Compound	Precision (RSD, n = 3)	
		Intra-Day	Inter-Day
**1**	Synephrine	0.75 *	1.63 *
		2.35 **	2.11 **
		2.79 ***	2.23 ***
**2**	Succinate	2.67 *	2.78 *
		1.41 **	1.74 **
		8.88 ***	8.88 ***
**3**	Liquiritin	0.84 *	3.86 *
		0.61 **	3.32 **
		0.95 ***	2.27 ***
**4**	Eriocitrin	1.93 *	4.16 *
		3.49 **	4.61 **
		4.07 ***	4.71 ***
**5**	Rutin	2.92 *	4.32 *
		1.08 **	4.65 **
		2.93 ***	3.00 ***
**6**	Narirutin	1.92 *	0.87 *
		3.15 **	2.65 **
		2.00 ***	4.06 ***
**7**	Naringin	1.07 *	2.98 *
		2.13 **	4.51 **
		1.60 ***	3.52 ***
**8**	Hesperidin	3.12 *	3.42 *
		0.67 **	3.73 **
		1.15 ***	2.56 ***
**9**	Neohesperidin	2.51 *	2.99 *
		1.22 **	3.16 **
		1.58 ***	2.83 ***
**10**	Liquiritigenin	1.42 *	2.22 *
		1.45 **	2.43 **
		1.21 ***	1.70 ***
**11**	Isoliquiritin	1.61 *	2.09 *
		1.11 **	3.04 **
		2.17 ***	2.58 ***
**12**	Didymin	0.21 *	4.62 *
		3.95 **	4.75 **
		1.02 ***	3.65 ***
**13**	Poncirin	1.46 *	2.40 *
		2.29 **	1.68 **
		1.94 ***	3.50 ***
**14**	6-Gingerol	1.63 *	2.27 *
		0.03 **	1.90 **
		2.05 ***	2.70 ***
**15**	Tangeretin	0.57 *	4.58 *
		0.12 **	4.65 **
		1.26 ***	4.14 ***
**16**	8-Gingerol	1.37 *	1.99 *
		1.18 **	2.67 **
		2.99 ***	2.85 ***
**17**	10-Gingerol	2.41 *	2.93 *
		1.14 **	2.26 **
		1.08 ***	2.14 ***
**18**	Pachymic acid	2.34 *	2.42 *
		1.83 **	1.70 **
		2.27 ***	2.41 ***
**19**	Dehydropachymic acid	4.98 *	2.73 *
		1.77 **	4.10 **
		7.85 ***	2.73 ***

* Low concentration; ** Medium concentration; *** High concentration.

**Table 4:** The range, the mean and standard deviation of the 19 chemical contents in a daily dose of WDD prepared in the lab and its five brands commercial preparations (μg).

Groups	A (n = 12)			B (n = 3)			C (n = 3)		
Compounds	Range	Mean	S.D.	Range	Mean	S.D.	Range	Mean	S.D.
Synephrine	1360.50–1957.75	1572.19	159.35	1093.58–1155.55	1117.77	33.14	2623.80–2728.26	2681.24	53.00
Succinate	470.03–699.63	568.02	72.84	270.78–299.66	285.69	14.46	768.73–800.35	782.46	16.22
Liquiritin	224.41–1813.21	907.48	596.41	3339.05–3813.76	3553.46	240.66	571.29–639.61	607.73	34.39
Eriocitrin	921.95–1579.45	1199.99	195.18	327.31–342.26	334.99	7.48	177.68–186.54	182.32	4.44
Rutin	353.30–915.77	615.34	180.55	225.73–269.04	245.99	21.79	349.59–388.10	368.29	19.28
Narirutin	79301.65–112193.03	93105.78	9112.75	32907.76–33507.87	33292.61	334.07	51895.32–52463.17	52090.53	322.84
Naringin	527.59–82105.92	67009.71	21502.32	33897.63–34121.30	33973.19	128.27	338.51–429.36	376.83	47.06
Hesperidin	29913.22–46665.13	40254.08	4700.37	19618.23–22825.16	21667.57	1779.75	15900.29–16613.15	16284.49	359.66
Neohesperidin	65657.36–91521.44	77906.35	9783.68	26406.45–31136.06	29446.21	2637.99	428.40–477.54	447.87	26.11
Liquiritigenin	41.37–243.06	109.61	58.36	437.76–444.82	442.38	4.00	405.04–410.87	407.80	2.93
Isoliquiritin	97.24–639.60	347.32	215.06	1471.90–1559.92	1521.89	45.21	269.10–321.15	293.68	26.15
Didymin	565.96–929.36	755.48	92.45	356.60–362.15	360.21	3.13	565.05–571.78	568.65	3.39
Poncirin	2322.15–3075.03	2666.62	200.88	5244.00–5164.11	5193.66	43.82	5146.47–5235.99	5194.92	45.21
6-Gingerol	128378.36–205274.14	156335.30	22480.96	16604.30–16863.58	16692.04	148.57	8961.68–9211.73	9124.51	141.14
Tangeretin	1129.54–1976.25	1648.56	231.20	5377.38–5436.15	5399.36	32.06	3466.61–3536.45	3504.87	35.40
8-Gingerol	5385.19–9595.66	7167.86	1268.38	1739.71–1740.92	1740.31	0.61	645.59–657.02	649.63	6.41
10-Gingerol	432.18–659.90	543.96	80.24	520.08–525.57	523.58	3.04	201.90–209.83	206.69	4.21
Pachymic acid	6.06–6.49	6.28	0.14	26.19–26.95	26.66	0.41	7.99–8.51	8.17	0.29
Dehydropachymic acid	7.66–12.85	9.09	1.45	41.98–53.36	48.85	6.04	11.73–13.90	12.77	1.09
Groups	D (n = 3)			E (n = 3)			F (n = 3)		
Compounds	Range	Mean	S.D.	Range	Mean	S.D.	Range	Mean	S.D.
Synephrine	3671.84–3861.10	3767.98	94.67	7060.75–7739.36	7357.05	347.38	4905.39–4944.79	4921.63	20.59
Succinate	251.84–300.97	279.85	25.28	676.16–717.40	694.22	21.09	413.42–478.03	456.30	37.13
Liquiritin	1015.33–1118.49	1059.25	53.26	984.20–1626.47	1222.36	351.82	13617.77–15724.51	14564.13	1069.55
Eriocitrin	290.66–305.08	295.51	8.29	383.18–406.50	395.93	11.81	1173.12–1210.99	1195.18	19.70
Rutin	541.15–569.97	555.15	14.43	695.88–992.91	806.83	162.14	1210.94–1296.73	1257.70	43.42
Narirutin	92828.60–93312.18	93021.54	256.16	130093.75–137452.04	133977.69	3696.20	172779.51–178910.51	175345.60	3185.21
Naringin	1413.32–1492.51	1448.57	40.30	159.93–244.43	212.06	45.58	209751.34–211319.50	210310.22	875.74
Hesperidin	40287.00–40996.44	40730.90	386.89	39769.45–49738.35	43461.55	5464.00	21120.41–31092.49	24460.55	5743.48
Neohesperidin	498.83–704.33	625.84	111.01	0.00	0.00	0.00	49188.87–72621.79	57826.56	12872.92
Liquiritigenin	96.74–116.63	107.44	10.03	243.92–259.92	249.45	9.07	4534.85–4611.06	4580.84	40.48
Isoliquiritin	500.57–581.77	531.68	43.80	455.05–749.51	558.83	165.35	17726.66–19734.03	19030.13	1130.05
Didymin	1072.58–1091.20	1084.19	10.13	3606.13–3680.67	3652.02	40.15	1361.67–1382.99	1375.17	11.74
Poncirin	10316.11–10510.81	10434.06	103.68	33187.91–36670.56	35475.26	1981.58	109082.60–112519.25	111174.26	1835.97
6-Gingerol	9334.53–9460.50	9390.02	64.31	24704.53–24813.85	24744.07	60.61	29095.47–29477.58	29229.93	214.73
Tangeretin	2636.84–2682.13	2658.65	22.69	13169.62–13408.28	13257.23	131.37	30155.63–30521.51	30370.05	190.89
8-Gingerol	2114.09–2144.77	2125.47	16.81	4756.19–4853.90	4802.04	49.13	3392.48–3515.84	3466.21	65.12
10-Gingerol	1444.69–1462.82	1454.12	9.09	2493.42–2569.45	2534.36	38.35	1111.32–1114.51	1112.73	1.63
Pachymic acid	18.44–21.28	19.49	1.56	41.33–45.14	43.31	1.91	25.87–30.12	28.66	2.41
Dehydropachymic acid	26.08–32.23	29.47	3.12	52.42–77.34	68.02	13.60	44.03–55.28	50.12	5.68

A: WDD prepared in the lab; B: WDD from Kotaro Pharmaceutical Co., Ltd.; C: WDD from Sheng Foong Co., Ltd.; D: WDD (concentrated particles) from Kaiser Pharmaceutical Co., Ltd.; E: WDD (concentrated ingots) from Kaiser Pharmaceutical Co., Ltd.; F: WDD from Sun Ten Pharmaceutical Co., Ltd.

## Supplementary Materials

The following are available online. Figure S1: MRM chromatogram of 19 standard chemicals. Figure S2: The characteristic product ion maps of the 19 chemicals. Figure S3: The four decoction pots in the WDD extraction process. Figure S4: Pinelliae Rhizoma (A), Bambusae Caulis in Taenias (B), Aurantii Fructus Immaturus (C), Citri Reticulatae Pericarpium (D), Glycyrrhizae Radix Et Rhizoma (E), Poria (F), Zingiberis Rhizoma (G) and Jujubae Fructus (H). Table S1: Chemicals contents in a daily dose of WDD prepared in the lab and its five brands commercial preparations (μg). Table S2: The proportion of the herbs and the excipiet in a daily dose of the WDD preparations. Table S3: The “total daily dosages of measured ingredient” for the 7 seven pieces of traditional Chinese medicine.
